# Rapid Onset and Resolution of Hydroxychloroquine Cardiomyopathy: A Case Report

**DOI:** 10.1155/2022/6503453

**Published:** 2022-09-26

**Authors:** Ahmad Ramahi, Amer Heider, J. Michelle Kahlenberg

**Affiliations:** ^1^Division of Rheumatology, Department of Internal Medicine, University of Michigan, Ann Arbor, MI 48103, USA; ^2^Department of Pathology, University of Michigan, Ann Arbor, MI 48103, USA

## Abstract

Systemic lupus erythematosus (SLE) is an autoimmune, chronic, and heterogenous disease with organ damage resulting from immune complex deposition and inflammatory infiltrates. Antimalarial drugs, such as hydroxychloroquine (HCQ), are cornerstone immunomodulators for the treatment of SLE. Rarely, HCQ toxicity can occur, leading to devastating outcomes. We report a case of a patient with SLE on HCQ who presented with a rapid onset of large pericardial effusion and a dramatically decreased left ventricular ejection fraction. Endomyocardial biopsy was positive for curvilinear bodies, confirming the diagnosis of hydroxychloroquine cardiotoxicity. Hydroxychloroquine cardiomyopathy is a rare but life-threatening medication side effect. It is important to consider it in any patient taking the medication who presents with a new onset or worsening symptoms of heart failure.

## 1. Introduction

Cardiotoxicity is a rare but serious side effect that should always be taken into consideration when evaluating patients on HCQ who develop new or worsening cardiac function. The diagnosis can be challenging, and multidisciplinary evaluation is recommended with endomyocardial biopsy and electron microscopic evaluation. Although we monitor our patients on HCQ for retinal toxicity, we do not have current guidelines to screen for cardiotoxicity. As HCQ blood level was found to predict HCQ retinopathy, further longitudinal prospective studies are needed to investigate the correlation between HCQ blood level and cardiotoxicity.

## 2. Case Presentation

A 41-year-old African American female with an 11-year history of stable postpartum cardiomyopathy (baseline ejection fraction of 30–35%) was referred to the rheumatology clinic due to a previous diagnosis of SLE.

Her rheumatic disease history extends two years backward when she developed bilateral finger pain, ulcers, and drainage. The patient was evaluated extensively; a transthoracic echocardiogram did not show evidence of infective endocarditis and an upper extremity angiogram showed scattered microocclusion of the bilateral fingers; flow did not respond to nitroglycerin. The patient developed a left index fingertip abscess for which incision and drainage were performed, followed by amputation.

Laboratory testing was notable for positive Antinuclear Antibody (ANA) by immunofluorescence assay (IFA) 1 : 1280 in a speckled pattern, positive Chromatin, SSA, Smith, and U1-RNP antibodies with elevated IgG levels of 2317 mg/dL. Furthermore, work up showed normal C3 and C4 complements with negative anti-dsDNA and anti-RNA polymerase III. The vasculitis workup was negative for antineutrophil cytoplasmic cntibodies (ANCA), antiproteinase-3 (PR3), antimyeloperoxidase antibodies (MPO), and cryoglobulins. Antiphospholipid antibodies including lupus anticoagulant, anti-beta-2-glycoprotein, and anticardiolipin were negative inflammatory markers were elevated; erythrocyte sedimentation rate (ESR) was 77 mm, and C-reactive protein (CRP) was 6.2 mg/dL. On physical examination, she had no skin thickening or other features of scleroderma.

Overall, our initial impression was of digital vasculitis secondary to systemic lupus erythematosus. The patient was started on hydroxychloroquine 300 mg daily (3.9 mg/kg/day), and she was advised to start mycophenolate mofetil. However, the patient chose to not start this medication, and she was lost to follow-up at our institution without a further discussion about other therapeutic options.

She represented to our institution one year later with significant weight loss from 227 to 135 bounds (l bs). Her physical examination showed progressive alopecia and a hypopigmented rash across her chest. Evaluation of her outside labs showed a progressive rise in her serum creatinine to 2.14 mg/dL from a baseline of 1.0 mg/dL. Repeated serologies showed low C3 (61 mg/dL), ESR 90 mm, and urine protein/creatinine 0.36. The serum hydroxychloroquine level was 1232.1 ng/ml (in SLE, the target blood hydroxychloroquine level is around 1000 ng/mL[1]). The ophthalmological exam at that time was normal. The patient was referred to nephrology and the hydroxychloroquine dose was dropped to 200 mg daily (3.1 mg/kg/day). Due to progressive kidney injury and a significant serologic profile of the patient, a kidney biopsy was conducted and showed vascular changes consistent with malignant hypertension and chronic thrombotic microangiopathy (TMA). Further testing, including pulmonary function testing, revealed a forced vital capacity (FVC) of 78%, forced expiratory volume 1 (FEV1) of 83%, and FEV1/FVC of 107% of predicted. The diffusing capacity for carbon monoxide (DLCO) was noted to be low, at 37% of the predicted. High-resolution computed tomography (HRCT) scan of the chest did not show evidence of interstitial lung disease (ILD). The course was further complicated by sepsis and acute on chronic kidney injury secondary to enteropathogenic E. coli in the stool. Additional workup showed ADAMTS 13 activity <5%, inhibitors 125%, and NT-ProBNP 995 pg/mL. Mycophenolate mofetil (MMF) was prescribed for systemic lupus with failure to thrive and chronic TMA secondary to the ADAMSTS 13 inhibitor.

Further workup for possible pulmonary hypertension was completed. A ventilation-perfusion (V/Q) scan showed no abnormalities, transthoracic echocardiography showed severe biventricular hypertrophy, and cardiac magnetic resonance imaging (MRI) showed no evidence of infiltrative cardiomyopathy. Her antihypertensive regimen was adjusted.

**Figure 1:**
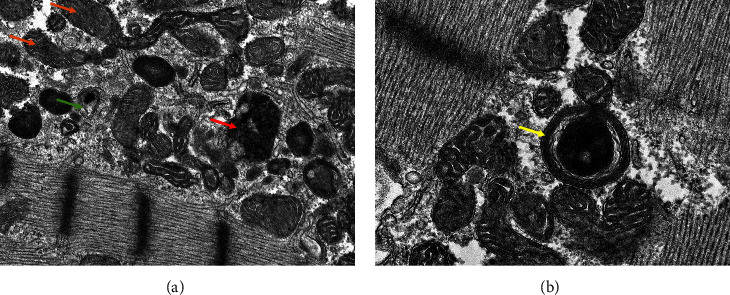
(a) Electron microscopic image of endomyocardial biopsy, 12k magnification, demonstrating elongated enlarged mitochondria (orange arrow), myelin figure (green arrow), and curvilinear body (red arrow). (b) Electron microscopic image of endomyocardial biopsy, 20k magnification, demonstrating myelin figure (yellow arrow).

Six months later, the patient continued to complain of shortness of breath and dyspnea with exertion. A repeat HRCT of the chest showed no interstitial changes but noted a new, large pericardial effusion for which the patient was admitted. The patient noted missing about 1.5 weeks of mycophenolate mofetil due to a lack of insurance coverage. Electrocardiogram (EKG) showed normal sinus rhythm with QT prolongation, QTc 527 ms, and a transthoracic echocardiogram showed worsening Left ventricular ejection fraction (LVEF) to 10%, pericardiocentesis yielded 860 ml of exudative serous fluid, cultures, and cytology were negative for infectious etiologies and malignancy. C3 was 67 and C4 was 17 mg/dL. Right heart catheterization with endomyocardial biopsy was done; no evidence of pulmonary hypertension was noted. Endomyocardial biopsy showed no features of myocarditis but demonstrated occasional curvilinear bodies with myelin figures and enlarged mitochondria (Figures 1(a) and 1(b)) consistent with hydroxychloroquine-induced cardiomyopathy on electron microscopy. The serum hydroxychloroquine level was 274 ng/ml at the time of biopsy. Hydroxychloroquine was stopped and the patient is currently being maintained on MMF and low-dose prednisone with improvement in her symptoms.

## 3. Discussion

We report a complex case of SLE characterized by lupus vasculitis that led to critical digital ischemia. This was followed by chronic TMA, which resulted in renal injury and pericarditis and HCQ cardiotoxicity. While the kidney biopsy showed features concerning scleroderma, the patient lacked other features of the disease. The presence of an ADAMTS13 inhibitor suggests a lupus-related TMA. It is possible that she exemplifies an overlap syndrome.

The immunomodulators have been used in treating SLE for a long time, of which the antimalarial drug and hydroxychloroquine (HCQ) is the cornerstone. The exact mechanism by which HCQ works is not completely understood, but proposed mechanisms include an increase in lysosomal pH, inhibition of lysosome-autophagosome fusion, binding to Toll-like receptor (TLR) agonists [2], and endosomal acidification. These changes interfere with Toll-like receptors 7 and 9 signaling in antigen-presenting cells and lead to decreased interferon-alpha production, a major contributor to SLE pathogenesis [3].

HCQ has been shown to be effective in treating SLE cutaneous disease and arthritis. HCQ also decreases thrombotic risk, improves pregnancy outcome in lupus patients, and lowers the flares risk, which in turn decreases steroid use and its associated accrual organ damage [4–6].

Although rare, HCQ can cause retinal toxicity, for which frequent retinal exams are recommended. Cardiomyopathy is a rarer side effect, for which we have no clear screening guidelines. HCQ cardiotoxicity has many manifestations, including conduction abnormalities like QT prolongation, incomplete or complete right or left bundle branch block, restrictive cardiomyopathy, and left ventricular hypertrophy with or without chamber dilatation [7]. The mechanism of HCQ cardiotoxicity is not well understood. Jordan et al., in their recently published study [8], showed that HCQ has ion channel blockade effect on cardiac myocytes. HCQ also decreases myocyte contractility by inhibiting sarcomere shortening, leading to a negative inotrope effect. Hypokalemia and hyperthermia were found to increase the incidence of HCQ-induced proarrhythmia [8]. Moreover, HCQ's effect on the autophagy-lysosome pathway leads to cytosolic accumulation of toxic metabolite products in the phagosomes, inhibiting lysosomal function, affecting mitochondria by decreasing oxidative phosphorylation, increasing mitochondrial DNA damage, and promoting apoptosis, leading to myonecrosis [9]. Another possible mechanism for HCQ cardiotoxicity is that HCQ binds directly to the myocyte membrane phospholipids, neutralizing phosphate groups and displacing calcium, leading to myofiber necrosis [9]. Risk factors for HCQ cardiotoxicity include long duration of therapy (more than 10 years), using higher doses per kilogram per day, female gender, older age, and previous cardiovascular or kidney disease [4]. Despite the short duration of HCQ treatment in our patient, her worsening renal function may have caused higher than desirable levels, leading to toxicity. Indeed, when her levels were examined, they were over 1200, which is a level at high risk for retinal toxicity [10]. Our patient became symptomatic over only a few weeks. Although it is not common it has been reported previously in the literature [11–13]. Although many patients may have risk factors for HCQ cardiotoxicity, it is not contraindicated to treat them with HCQ due to the aforementioned HCQ benefits as long as they take the recommended dose and are actively monitored for potential complications. Our patient's initial poor adherence and decision not to proceed with further immunosuppression put her at a higher risk for this complication. Her uncontrolled disease due to lack of immunosuppression led to kidney injury, and as the patient was not being actively monitored by her outside physician, her HCQ dose was not really adjusted which led to this complication.

Diagnosing HCQ cardiomyopathy is challenging, especially in patients with a preexisting cardiac condition, like our patient. Echocardiogram features may be suggestive but not diagnostic. Endomyocardial biopsy showing sarcoplasmic myelinoid and curvilinear bodies on electron microscopy are pathognomonic [4]. In our patient, curvilinear bodies with myelin figures and enlarged mitochondria were observed. The outcome of HCQ cardiac toxicity varies, ranging from complete resolution to death, with the more severe outcomes being noticed in those with the longest duration of HCQ treatment [13]. Our patient improved after HCQ discontinuation, and her LVEF returned to her baseline of 30–35% after 3 months.

## 4. Conclusions

Cardiotoxicity is a rare but serious side effect that should always be taken into consideration when evaluating patients on HCQ who develop new or worsening cardiac function. The diagnosis can be challenging, and multidisciplinary evaluation is recommended with endomyocardial biopsy and electron microscopic evaluation. Although we monitor our patients on HCQ for retinal toxicity, we do not have current guidelines to screen for cardiotoxicity. A yearly EKG may be advisable for patients at risk. As HCQ blood level was found to predict HCQ retinopathy, further longitudinal prospective studies are needed to investigate the correlation between HCQ blood level and cardiotoxicity.

## Data Availability

No data were used in the preparation of this case report.
